# Indirect Self-Destructiveness and Emotional Intelligence

**DOI:** 10.1007/s11126-015-9387-x

**Published:** 2015-07-12

**Authors:** Konstantinos Tsirigotis

**Affiliations:** Department of Psychology, The Jan Kochanowski University in Kielce, Piotrków Trybunalski Branch, Słowackiego 114/118 str., 97-300 Piotrków Trybunalski, Poland

**Keywords:** Indirect self-destructiveness, Emotional intelligence, Mental health, Psychological well-being

## Abstract

While emotional intelligence may have a favourable influence on the life and psychological and social functioning of the individual, indirect self-destructiveness exerts a rather negative influence. The aim of this study has been to explore possible relations between indirect self-destructiveness and emotional intelligence. A population of 260 individuals (130 females and 130 males) aged 20–30 (mean age of 24.5) was studied by using the Polish version of the chronic self-destructiveness scale and INTE, i.e., the Polish version of the assessing emotions scale. Indirect self-destructiveness has significant correlations with all variables of INTE (overall score, factor I, factor II), and these correlations are negative. The intensity of indirect self-destructiveness differentiates significantly the height of the emotional intelligence and vice versa: the height of the emotional intelligence differentiates significantly the intensity of indirect self-destructiveness. Indirect self-destructiveness has negative correlations with emotional intelligence as well as its components: the ability to recognize emotions and the ability to utilize emotions. The height of emotional intelligence differentiates the intensity of indirect self-destructiveness, and vice versa: the intensity of indirect self-destructiveness differentiates the height of emotional intelligence. It seems advisable to use emotional intelligence in the prophylactic and therapeutic work with persons with various types of disorders, especially with the syndrome of indirect self-destructiveness.

## Introduction

Emotions are an important group of psychological processes which influence entire psychological life and psychological functioning of the man. In the history of the philosophical and psychological thought two currents or previews were clashing: some authors believed that the man is motivated primarily by emotional processes, and others, that by cognitive or intellectual processes. Until recently, in the Western tradition of thinking about the psychological life emotions were regarded mainly as a factor disrupting intellectual processes. Only in the second half the Twentieth century appeared hypotheses, that emotions could have a positive effect on intellectual processes and psychological functioning in general [1].

The construct of emotional intelligence has been formed as a result of an attempt at answering the question as to why some people are better than others at maintaining psychological wellbeing. For a long time, studies into intelligence were dominated by cognitive intelligence, although some researchers [2] drew attention to the fact that individuals having a high intelligence quotient (IQ) are not always efficient at coping with ordinary, everyday life and psychological tasks, while other individuals, with a lower IQ, come out very well at the same tasks. There is a view that it is differences in emotional intelligence that may be responsible for those discrepancies between cognitive intelligence and social functioning. The importance of emotional intelligence can be demonstrated, among others, by the idea thought up by researchers in the field of artificial intelligence to “add” emotions to computers in order to prioritize and direct their activity [1].

According to Salovay and Mayer’s model, emotional intelligence is a set of abilities and a subset of social intelligence that includes the following three categories of adaptive abilities: appraisal and expression of emotions, regulation of emotions and utilization of emotions in problem solving. The first category consists of components of appraisal and expression of own emotions and appraisal of emotions of others. The component of appraisal and expression of own emotions is further divided into two subcomponents, i.e.: verbal and non-verbal, while the component of appraisal of emotions of others is divided into subcomponents of non-verbal perception and empathy. The second category of emotional intelligence—regulation—includes components of regulation of emotions in self and regulation of emotions in others. The third category—utilization of emotions—incorporates components of flexible planning, creative thinking, redirected attention and motivation. Even though emotions are at the core of the model, it also includes social and cognitive functions connected with expression, regulation and utilization of emotions [1, 3]. Mayer et al. [4] further developed that model, but in the opinion of many authors, fundamental aspects of emotional intelligence proposed in the latest model are similar to those contained in the 1990 one [5].

Consequently, individuals who have developed abilities connected with emotional intelligence understand and express their own emotions, recognize emotions of others, regulate affect and utilize moods and emotions to motivate adaptive behaviours [1]. Authors wonder whether it is not yet another definition of a healthy, self-actualizing individual.

Moreover, authors notice relationships between emotional intelligence and health. According to them, an emotionally intelligent individual can be considered to be such that has achieved at least a certain form of positive mental health. Such individuals are aware of their own and others’ feelings. They are open to positive and negative aspects of internal experience, able to name them and communicate them when needed. Such awareness often leads to the effective regulation of one’s own emotions and emotions of others, hence contributing to wellbeing [1].

Studies proved that individuals of higher emotional intelligence have a tendency towards positive mood and are more capable of improving their mood after negative one [6, 7]. Generally speaking, higher emotional intelligence is connected with better psychophysical health [7].

Emotional intelligence is associated with direct functioning, while cognitive intelligence is connected with long-term strategic competence. In other words, emotional intelligence is process- rather than result-oriented [2, 8].

A majority of authors usually consider “self-destructive behaviours” to be behaviours categorised as directly self-destructive, most frequently self-mutilation, self-inflicted injury, and attempted or committed suicide. Literature usually offers studies into direct self-destructiveness (self-mutilation, self-inflicted injury, attempted suicide, committed suicide) or into specific and separate behaviours being manifestations of what is nowadays called indirect or chronic self-destructiveness.

While the issue of directly self-destructive behaviours (suicides, self-inflicted injuries etc.,) is clear and raises no doubt, less acute and “subtle” forms of self-harm or impairing the quality and/or shortening the length of one’s life are not immediately and directly noticeable (e.g., risky behaviours, addictions, neglects etc.). Less attention is usually paid to them, especially as numerous of those are treated as commonly (or at least often) occurring behaviours, and thus “normal” ones.

Kelley describes chronic self-destructiveness as a generalised tendency to undertake behaviours increasing the probability of negative and decreasing the probability of positive consequences for the subject [9]. For the purposes of this study, it was assumed that indirect/chronic self-destructiveness comprises behaviours whose probable negative effect is intermediated by additional factors, while the relationship between a behaviour and harm is perceived as probable. Indirect self-destructiveness understood in such a way includes both taking and abandoning specific actions; it concerns getting into hazardous and increased-risk situations (active form) or neglecting one’s safety or health (passive form). Moreover, indirect self-destructiveness is a form of self-destruction characterised by an increased temporal distance between an action and its effect [10, 11]. There are, in general, several categories of indirectly self-destructive behaviours: transgression and risk, poor health maintenance, personal and social neglects, lack of planfulness, and helplessness and passiveness when facing problems/difficulties. Indirect self-destructiveness includes among others risky behaviours undertaken for a momentary pleasure, e.g., driving with bravado connected with a desire to impress others, feel appreciated, better or noticed, or gambling, succumbing to temptations, impulsiveness, and seeking risky excitation [9–11].

Researches have shown that individuals who are primarily motivated by current emotional factors are more likely than those motivated by more distant cognitive considerations to engage in acts that are ultimately self-destructive. Generally, those individuals who are more responsive to immediate emotional factors than to more distant rational projections of consequences are likely to engage in maladaptive acts. Though the specific acts in question vary widely, the unifying characteristic seems to be response to affect rather than to cognitions. Each behaviour appears to represent the tendency to seek immediate pleasure or avoid immediate discomfort, regardless of the long-term consequences of doing so [9].

While emotional intelligence may have a favourable influence on the life and psychological and social functioning of the individual, indirect self-destructiveness exerts a rather negative influence. World literature offers almost no studies into relations between indirect self-destructiveness and emotional intelligence.

The aim of this study has been to explore possible relations between indirect self-destructiveness and emotional intelligence.

## Methods

The study is part of two more extensive research projects (on indirect self-destructiveness and on emotional intelligence) and thus the applied methodology or some other parts may be similar.

## Participants

A population of 260 individuals (130 females and 130 males) aged 20–30 (mean age of 24.5) was studied by using the Polish version of the chronic self-destructiveness scale (CS-DS) by Kelley et al. [9], in its adaptation by Suchańska [10] and the Polish version of assessing emotions scale (AES) by Schutte et al. [3] in its adaptation by Ciechanowicz, Jaworowska and Matczak [12]. The study group was constructed on the basis of random selection from the general population (of healthy subjects); participation in the study was voluntary and anonymous and according to the principles of the Declaration of Helsinki.

## Materials

In order to examine indirect (chronic) self-destructiveness as a generalised tendency, Kelley created a research tool comprising several categories of indirectly self-destructive behaviour; the ultimate version was made up of a Likert-type internally consistent set of 52 items with the total obtained score informing about the intensity of indirect self-destructiveness. Both the original scale and its Polish adaptation are characterised by high reliability and validity [9, 10].

Schutte et al. [3] created the tool to examine emotional intelligence. Since then, the questionnaire has been used in many studies, but under different names: “Emotional Intelligence Scale” (EIS) [5, 13, 14], “Schutte Self-Report Inventory for Emotional Intelligence” (SSRI) [15] and “Schutte Emotional Intelligence Scale” (SEIS) [5, 16]. That has most probably resulted from the fact that the authors of the tool did not give it a name on its creation [3]! They only mentioned “emotional intelligence scale” [3, p. 175], although as a common rather than proper name. They first used the “Assessing Emotions Scale” (AES) name in later studies [5, 7]. This study applies the Emotional Intelligence Questionnaire INTE, i.e., the Polish version of AES, as adapted by Ciechanowicz, Jaworowska and Matczak [12]. The questionnaire is composed of 33 items on which the subject may take a position by choosing one of the five possible answers (the Likert type scale). Along with the general emotional intelligence score, the scale enables to receive scores on two factors: factor I is ability to utilize emotions in order to support thinking and actions, while factor II is ability to recognize emotions. Both the American and Polish versions are characterized by high reliability and validity [3, 12].

## Statistical Analysis

The statistical analysis of received scores applied descriptive methods and statistical inference methods. In order to describe the mean value for quantitative traits, the arithmetic mean (M) was calculated, while the standard deviation (SD) was assumed to be the dispersion measure. The conformity of distributions of quantitative traits with the normal distribution was assessed using the Shapiro–Wilk test. Due to the lack of conformity of distributions of dependent variables with the normal distribution, the statistical processing of received results used non-parametric statistics. In order to examine the relationship between the studied variables Kendall’s “tau” (*τ*) correlation coefficient was used. Hierarchical cluster analysis was used to extract clusters (groups) of the subjects with the most similar (close to each other) results in the studied variables. Analysis of Variance (ANOVA) and post hoc comparisons using the Tukey’s HSD (Honestly Significant Difference) test for unequal N were applied in order to explore the differences of scores in the individual clusters. For all the analyses, the maximum acceptable type I error was assumed at α = 0.05. Asymptotic two-sided test probability p was calculated and p ≤ 0.05 was considered statistically significant. The statistical analyses were performed by means of the *Statistica PL 10.0 for Windows* [17] statistical package.

## Results

The mean scores of the participants in the variables measured by both tools were in the range of average results. The CS-DS mean was 113.070 (SD 19.506), in the INTE mean: 5.103 (SD 1.743), in factor I of INTE mean 5.492 (SD 1.538), and in factor II of INTE mean: 5.032 (SD 1.893).

**Fig. 1:**
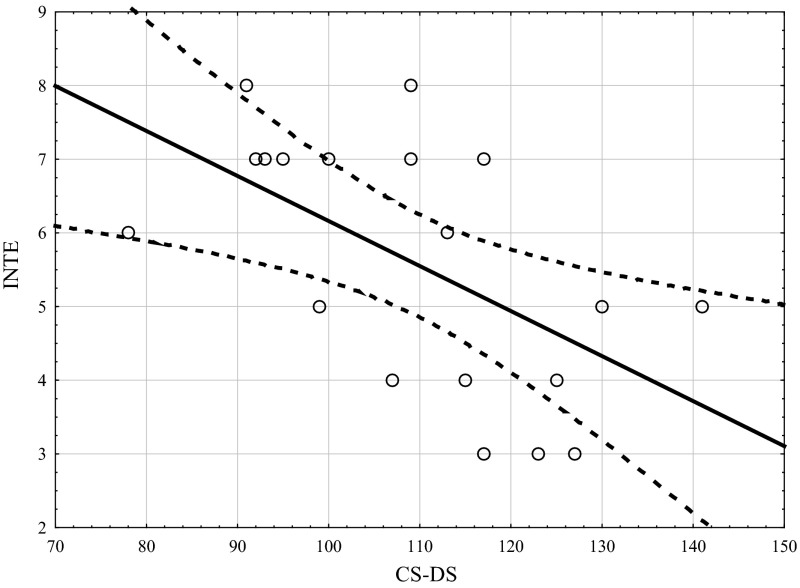
Scatterplot matrix of variables scores (INTE, CS-DS). *INTE* polish version of the “Assessing Emotions Scale”. *CS-DS* polish version of the “Chronic Self-Destructiveness Scale”

**Fig. 2:**
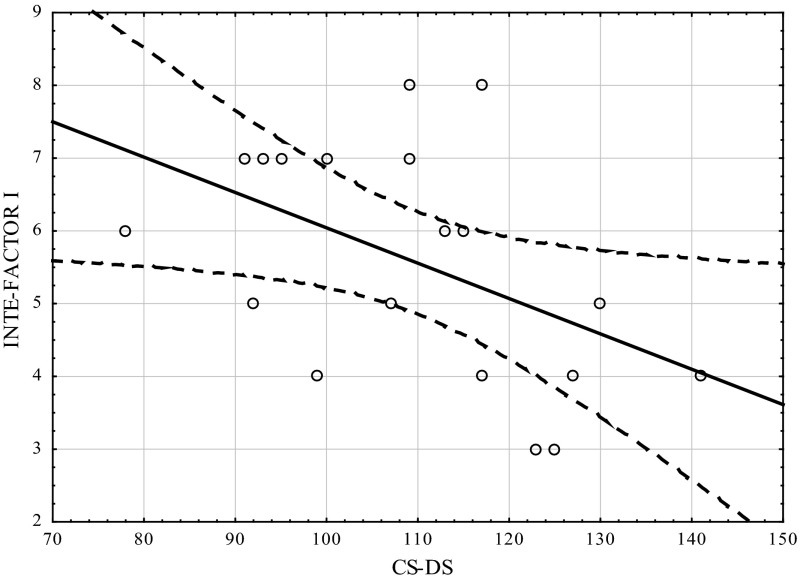
Scatterplot matrix of variables scores (INTE-Factor I, CS-DS). *INTE-Factor I* ability to utilize emotions. *CS-DS* polish version of the “Chronic Self-Destructiveness Scale”

**Fig. 3:**
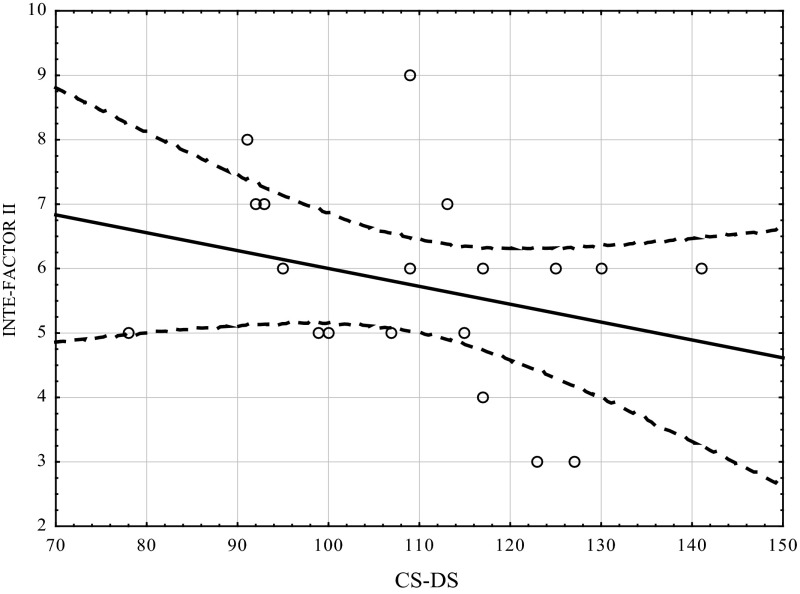
Scatterplot matrix of variables scores (INTE-Factor II, CS-DS). *INTE-Factor II* ability to recognize emotions. *CS-DS* polish version of the “Chronic Self-Destructiveness Scale”

Table 1 shows the correlation coefficients (Kendall’s tau) between the studied variables using the CS-DS and INTE. Figures 1, 2, 3 show the scatterplot matrices of these scores. As can be seen indirect self-destructiveness has significant correlations with all variables of INTE (overall score, factor I, factor II), and these correlations are negative (Table 2).

**Table 1:** Correlation coefficients between variables measured by CS-DS and INTE

Variable	INTE	Factor I	Factor II
CS-DS	−0.605 *p* = 0.0004	−0.522 *p* = 0.002	−0.309 *p* = 0.01

*CS-DS* polish version of the “Chronic Self-Destructiveness Scale”, *INTE* polish version of the “Assessing Emotions Scale”, *Factor I* ability to utilize emotions, *Factor II* ability to recognize emotions

**Table 2:** Hierarchical cluster analysis of CS-DS

Variable	M (CS-DS)	SD	n	INTE
Cluster 1 (low CS-DS)	94.940	8.047	100	5.816
Cluster 2 (medium CS-DS)	117.468	7.181	124	4.706
Cluster 3 (high CS-DS)	148.278	12.498	36	4.633

*CS-DS* polish version of the “Chronic Self-Destructiveness Scale”, *INTE* polish version of the “Assessing Emotions Scale”

For a deeper exploration of the relationship between the studied variables, hierarchical cluster analysis and analysis of variance (ANOVA) were performed. Table 3 presents data on the clusters extracted in the CS-DS. As can be seen participants, in terms of the similarity of their scores have been grouped into three clusters: cluster 1 with low scores in CS-DS (94.940), cluster 2 with medium scores (117.468) and cluster 3 with high scores (148.278); the most numerous is the cluster with medium scores (124 subjects), and the least numerous is the one of high scores (36 subjects). The same table shows that the ratio of scores in CS-DS to INTE scores is inversely proportional: the higher the score in CS-DS, the lower the score in INTE (and vice versa: the lower the score in CS-DS, the higher the score in INTE). In order to detect statistically significant differences between the results obtained in INTE by subjects qualified to individual clusters of CS-DS, analysis of variance (ANOVA) and “post hoc” comparisons (Table 4) were performed. The analysis of variance shows that the intensity of indirect self-destructiveness differentiates significantly the height of the emotional intelligence (F = 7.850, *p* = 0.0006); “post hoc” comparisons show that subjects with low scores in the CS-DS have significantly higher scores in INTE than those in the other two clusters.

**Table 3:** “Post-hoc” comparisons of INTE regarding clusters of CS-DS

Tukey’s HSD test for unequal N; variable: INTE			
Cluster of CS-DS	(Low) 5.816	(Medium) 4.706	(High) 4.633
Low (5.816)	–	*p* = 0.0006	*p* = 0.04
Medium (4.706)	*p* = 0.0006	–	ns
High (4.633)	*p* = 0.04	ns	–

*INTE* polish version of the “Assessing Emotions Scale”, *CS-DS* polish version of the “Chronic Self-Destructiveness Scale”, *HSD* honestly significant difference

**Table 4:** Hierarchical cluster analysis for INTE

Variable	M (INTE)	SD	*n*	CS-DS
Cluster 1 (low INTE)	3.048	0.961	86	118.600
Cluster 2 (medium INTE)	5.509	0.505	110	112.400
Cluster 3 (high INTE)	7.193	0.477	64	106.030

*INTE* polish version of the “Assessing Emotions Scale”, *CS-DS* polish version of the “Chronic Self-Destructiveness Scale”

In order to examine differences in the CS-DS according to the results of INTE a similar exploratory strategy was used. Table 4 presents data of the hierarchical cluster analysis of subjects’ scores in INTE. As can be seen the subjects, in terms of the similarity of their scores have been grouped into three clusters: cluster 1 with low scores in INTE (3.048), cluster 2 with medium scores (5.509) and cluster 3 with high scores (7.193); the most numerous cluster is with medium scores (110 subjects), and the least numerous is the cluster with high scores (64 subjects). The same table shows that the ratio of scores in INTE to CS-DS scores is inversely proportional: the higher the score in INTE, the lower the score in CS-DS (and vice versa: the lower the score in INTE, the higher the score in CS-DS). In order to detect statistically significant differences between the results obtained in CS-DS by subjects qualified to individual clusters of INTE, analysis of variance (ANOVA) and “post hoc” comparisons (Table 5) were performed. The analysis of variance shows that the height of the emotional intelligence differentiates significantly the intensity of indirect self-destructiveness (F = 3968, *p* = 0.02); “post hoc” comparisons show that subjects with high scores in INTE have significantly lower scores in CS-DS than those in the cluster of low scores in INTE.

**Table 5:** “Post-hoc” comparisons of INTE regarding clusters of INTE

Tukey’s HSD test for unequal *N*; variable: CS-DS			
Cluster of INTE	(High) 118.600	(Medium) 112.400	(High) 106.030
Low (118.600)	–	ns	*p* = 0.01
Medium (112.400)	ns	–	ns
High (106.030)	*p* = 0.01	ns	–

*CS-DS* polish version of the “Chronic Self-Destructiveness Scale”, *INTE* polish version of the “Assessing Emotions Scale”, *HSD* honestly significant difference

The results of these analyzes confirm the results of the negative correlations between CS-DS and INTE: the higher scores in the INTE, the lower scores in the CS-DS and vice versa: the lower scores in the INTE, the higher scores in the CS-DS.

## Discussion

In discussing the results, it will be difficult to refer to the results of other research in this area, because there were not found in the available literature works dealing with this issue.

The results of the participants in all the measured variables are within the range of mean results, so it can be expected that some serious deviations (on the plus or minus) in indirect self-destructiveness or emotional intelligence does not affect the shaping of studied phenomena.

The authors of one of the pioneering and most well-known concept of emotional intelligence are asking the question whether a person with high emotional intelligence is not a healthy, self-actualized one? In other words, isn’t the concept of emotional intelligence another definition of a healthy, self-actualized person? [1]. This finding is consistent with the results of many studies, including in the present work.

Emotional intelligence negatively correlates, among others, with deviant social behaviour (active form of indirect self-destructiveness) and depression, feelings of hopelessness and helplessness (passive form of indirect self-destructiveness), anxiety and suicidal ideation [18]. In turn, the occurrence of associations between suicide attempts and indirect self-destructiveness (its severity and manifestations as well) has been shown in other studies [19–22].

Another research team [23] reported the possibility of prediction, and prevention of disorders of adaptation, such as aggression, violence and drug abuse. Aggression and violence and drug abuse are even textbook manifestations of indirect self-destructiveness, particularly its active form (risky and transgressive behaviour). Aggression and violence allow for the immediate discharge of anger or other unpleasant (the so-called negative) emotions or to achieve some other goal (instrumental aggression/violence); use of psychoactive substances, in turn, results in change of perception/mood, euphoria or excitement and study results confirm the occurrence of the relationship between psychoactive substance use and indirect self-destructiveness [24]. We are dealing here with a quick, even instantaneous gratification, mainly of emotional nature.

One of the manifestations of indirect self-destructiveness, as mentioned above, is impulsivity and impulsive behaviour. And here the results of other studies are in line with the results of the present work. Emotional intelligence is associated with better control of impulses [5, 7, 25, 26] and, vice versa, low emotional intelligence is associated with less control of impulses [3, 7]. In addition, the lack of awareness of emotions and inability to control them are the main symptoms of certain types of disorders not only of impulse control, but even personality [7, 26]. Impulsive behaviours are, or very easily become, risk behaviours.

Impulse control problems are very common in the case of psychopathy or psychopathic personality or dissocial personality (ICD-10) or antisocial personality (DSM-5). Regardless of the naming, the authors of the concept and classification all agree on the fact that the behavior of psychopaths is harmful and even destructive to other people. But regardless of the fact that behavior of psychopaths is destructive to other people they are also self-destructive, but indirectly: succumbing to temptations, impulsiveness, desire for immediate gratification, aggressiveness. It was found that psychopathic individuals with a high intensity of anxiety (“secondary psychopaths”) are significantly lower in emotional intelligence than psychopaths with low intensity of anxiety (“primary psychopaths”) and those without psychotic disorders [27].

Dramatic manifestation of the previously mentioned psychological dysfunctions and dysfunction in interpersonal relations is domestic violence. It found that the perpetrators of domestic violence (men) have lower emotional intelligence than the general population. Besides, deficits in emotional intelligence are associated with a tendency for violence in both the perpetrators of violence as well as in the general population [8].

Some authors [28] suggest to call emotional intelligence “emotional self-efficacy”. And self-efficacy is the opposite, and even negation of self-handicapping, one of the main components of the indirect self-destructiveness, especially its passive form.

Emotional intelligence is associated with greater optimism, lack of depressive states [3], greater empathy and greater self-control in social situations [5, 25]. Empathy and self-control in social situations are the opposite of two categories of indirect self-destructiveness: impulsivity and social neglects.

A person with high emotional intelligence is less likely to engage in problem behaviors and avoids negative and self-destructive behavior such as smoking, alcohol abuse, drug use or violence [4].

Generally it can be said that low emotional intelligence is associated with poor psychosocial functioning [7], which in turn is associated with indirect self-destructiveness as generalized behavioural tendency.

Aforementioned Bar-On believes that emotional intelligence relates to immediate functioning [2]. And that immediate functioning can be favourable or unfavourable, as in the case of indirect self-destructiveness, in which it is more important for the individual the direct (or immediate) or quick gratification than the long-term effects, mostly negative ones.

Having accepted that the syndrome of indirect self-destructiveness is a type (kind of) psychological dysfunction, it is easy to understand the meaning of above presented results and statements: indirect self-destructiveness is not conducive to emotional intelligence (and possibly even disturbs it), and emotional intelligence is a protective factor against the indirect self-destructiveness. Emotional intelligence can be seen as one of the psychological resources conducive to well-being, especially the psychological one.

The statements above are in line with the results of a meta-analysis, according to which higher emotional intelligence is associated with better mental, psychosomatic and physical health [7].

In conclusion, it is worth quoting the words of the authors of emotional intelligence concept, who said simply that people who do not learn to regulate their own emotions may become slaves to them [1].

The prophylactic and therapeutic work with persons with various types of disorders, especially with the syndrome of indirect self-destructiveness, should also take into account emotional intelligence.

## Conclusions

Indirect self-destructiveness, as a generalised behavioural tendency, has negative correlations with emotional intelligence as well as its components: the ability to recognize emotions and the ability to utilize emotions. The height of emotional intelligence differentiates the intensity of indirect self-destructiveness, and vice versa: the intensity of indirect self-destructiveness differentiates the height of emotional intelligence. It seems advisable to use emotional intelligence in the prophylactic and therapeutic work with persons with various types of disorders, especially with the syndrome of indirect self-destructiveness.
